# Prior Bariatric Surgery Predicts Lower Life-Threatening Morbidity in Patients Admitted for Acute Allergic Reaction and Anaphylaxis: a Propensity Score-Matched Analysis of the U.S. Nationwide Inpatient Sample, 2005–2018

**DOI:** 10.1007/s11695-024-07115-6

**Published:** 2024-07-24

**Authors:** Po-Chun Wang, Wei-Ning Lin

**Affiliations:** 1 PhD Program in Nutrition and Food Science, Fu Jen Catholic University, New Taipei City, Taiwan; 2 Department of General Surgery, Mackay Memorial Hospital, Taipei, Taiwan; 3 Graduate Institute of Biomedical and Pharmaceutical Science, Fu Jen Catholic University, New Taipei City, Taiwan

**Keywords:** Acute allergic reaction, Anaphylaxis, Bariatric surgery, Nationwide Inpatient Sample (NIS), Obesity

## Abstract

**Purpose:**

Acute allergic reactions may occur in susceptible individuals following exposure to various allergens. Obesity is linked to allergic reactions, and weight loss from bariatric surgery may attenuate the severity of certain conditions such as airway hyperresponsiveness in asthma. This retrospective observational study investigates associations between prior bariatric surgery and lower risk for life-threatening conditions in patients hospitalized with acute allergic reactions and anaphylaxis.

**Materials and Methods:**

Adults ≥ 18 years old diagnosed with morbid obesity and admitted to US hospitals with acute allergic reactions/anaphylaxis were included. All data were extracted from the US Nationwide Inpatient Sample (NIS) database 2005–2018. Patients without information on in-hospital mortality, discharge destination, hospital costs, and length of stay (LOS) were excluded. Patients were divided into two groups based on prior bariatric surgery or not. All diagnoses were verified through ICD-9 and ICD-10 codes. Between-group differences and associations between variables were evaluated using logistic regression analysis.

**Results:**

After matching, patients with prior bariatric surgery had significantly lower proportions of any life-threatening morbidity (37.2% vs. 47.4%), respiratory distress or failure (11.2% vs. 17.0%), pneumonia or severe infection (7.4% vs. 10.2%), sepsis/septic shock (15.2% vs. 20.9%), intubation and mechanical ventilation (11.2% vs. 14.6%), prolonged LOS (10.3% vs. 20.6%) and unfavorable discharge (6.9% vs. 12.5%) than those without prior bariatric surgery.

**Conclusion:**

Prior bariatric surgery predicts a lower risk of life-threatening morbidity and prolonged LOS among adults hospitalized for acute allergic reaction and anaphylaxis. Future prospective studies are warranted to confirm the present findings and reveal underlying mechanisms.

**Graphical Abstract:**

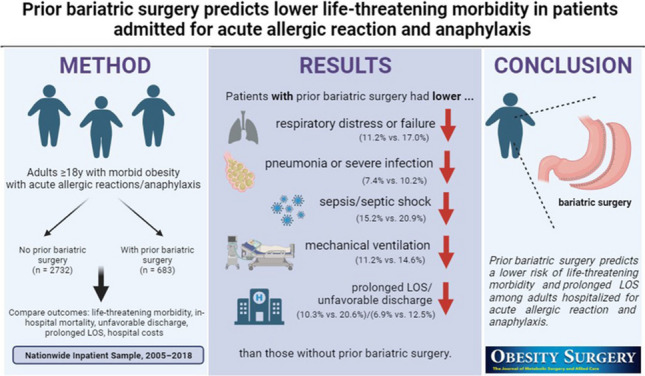

**Supplementary Information:**

The online version contains supplementary material available at 10.1007/s11695-024-07115-6.

## Introduction

Acute allergic reactions may occur following exposure to various allergens such as foods, medications, biologic compounds, plants, or venoms, and in a broad range of settings like homes, schools, or hospitals. Reactions are graded on a severity continuum ranging from mild (requiring no or minimal intervention) to anaphylactic shock (requiring treatment with resuscitative therapies) [1]. Acute allergic reactions, including anaphylaxis, place a heavy burden on patients, their families, and the healthcare system. In the US, from 2008 to 2016, more than 400,000 emergency visits were recorded for anaphylaxis alone [2]. Previous studies have reported increasing incidence of severe allergic reactions and anaphylaxis, and symptoms of anaphylaxis may present rapidly, often followed by airway compromise, respiratory distress, hemodynamic instability, and, potentially, death [3].

Recent studies have suggested a link between obesity and allergic reactions, including drug allergies and food allergies, particularly immediate responses [4, 5]. At the same time, strong evidence has verified the efficacy and safety of modern bariatric procedures [6], while also showing that weight loss obtained through bariatric surgery attenuates the severity and adverse outcomes of various diseases, including autoimmune diseases and airway hyperresponsiveness in asthma [7–10].

Bariatric surgery is associated with a reduced incidence of new diabetes, hypertension, and hypercholesterolemia, ensuring prolonged weight loss and improving patients’ quality of life [11, 12]. Globally, around 580,000 people undergo bariatric surgery annually [13]. The American Society for Metabolic and Bariatric Surgery (ASMBS) estimated that in 2019, more than 256,000 individuals in the United States underwent bariatric surgery [11]. However, to date, no study has evaluated the potential benefits of bariatric surgery in patients admitted for acute allergic reaction and anaphylaxis. We hypothesized that prior bariatric surgery may be associated with a lower risk of life-threatening conditions in patients hospitalized with allergic reactions and anaphylaxis. Therefore, this study aimed to determine the influence of prior bariatric surgery on hospitalization outcomes of patients with acute allergic reactions and anaphylaxis using a large, nationally representative database.

## Material and Methods

### Study Design and Data Source

This population-based, retrospective observational study extracted all data from the US Nationwide Inpatient Sample (NIS) database, the largest all-payer, continuous inpatient care database in the United States, including about 8 million hospital stays each year (Healthcare Cost and Utilization Project [HCUP]. *Introduction to the Nationwide Inpatient Sample* [NIS]. Rockville, MD: Agency for Healthcare Research and Quality; 2008.). HCUP, a division of the US National Institutes of Health (NIH) administers the database. Patient data include primary and secondary diagnoses, primary and secondary procedures, admission and discharge status, patient demographics, expected payment source, duration of hospital stay, and hospital characteristics (i.e., bed size/location/teaching status/hospital region). All admitted patients are initially considered for inclusion. The continuous, annually updated NIS database derives patient data from about 1,050 hospitals in 44 states in the US, representing a 20% stratified sample of US community hospitals as defined by the American Hospital Association.

### Ethics Statement

All data used in this study were obtained through request to the Online HCUP Central Distributor. This study conforms to the NIS data-use agreement with HCUP. The study was exempted from Institutional Review Board (IRB) approval because there was no direct involvement by patients or other persons as the study used secondary data from the NIS database. Because all data in the NIS database are de-identified, the requirement of informed consent was also waived.

### Study Sample Selection

This study utilized the International Classification of Diseases, Ninth Revision (ICD-9) and Tenth (ICD-10) diagnostic codes to identify adults ≥ 18 years old with a diagnosis of morbid obesity (ICD-9: 278.01, V85.35-V85.45; ICD-10: E66.01, Z68.4x) and who were admitted to US hospitals with a primary diagnosis of acute allergic reactions/anaphylaxis between 2005 and 2018 as recorded in the NIS database. The patients were further divided into two groups based on whether or not they had prior bariatric surgery (laparoscopic or open Roux-en-Y gastric bypass, laparoscopic adjustable gastric band, and laparoscopic sleeve gastrectomy) through ICD-9 and ICD-10 codes, as shown in previous studies [14]. Patients with no information on in-hospital mortality, discharge destination, hospital costs, and length of stay (LOS) were excluded. The ICD codes for identifying acute allergic reactions/anaphylaxis and bariatric surgery are listed in Supplementary Table 1.

### Study Variables and Outcome Measures

Study endpoints were: incidence of 1) any life-threatening morbidity that occurred during admission; 2) in-hospital mortality; 3) unfavorable discharge, defined as transfer to nursing homes or long-term care facilities; 4) prolonged LOS, defined as LOS >  = 75th percentile; and 5) hospital costs. Significant morbidities included cardiac arrest and dysrhythmia, acute myocardial infarction (AMI) and cerebrovascular accident (CVA), venous thromboembolism (VTE), respiratory distress or failure, pneumonia, and severe infection, sepsis and septic shock, and intubation and mechanical ventilation. The ICD codes for identifying these morbidities are listed in Supplementary Table 1.

#### Covariates

Patients’ demographic characteristics included age, sex, race, household income, and insurance status (primary payer). Clinical characteristics included significant comorbidities (i.e., coronary artery disease, congestive heart failure, diabetes, hypertension, cerebrovascular disease, chronic respiratory disease, rheumatic disease, and renal insufficiency), which were identified using ICD codes. Hospital-related characteristics (bed size, location/teaching status, hospital region) were also extracted from the database as part of the comprehensive data available for all participants.

### Statistical Analysis

All analyses were performed using SAS survey statements to address the complex sampling design for HCUP-NIS data (SAS Institute Inc., Cary, NC, USA). Continuous variables are presented as weighted mean and standard error (SE); categorical variables are presented as unweighted numbers and weighted proportions. Differences in means between the groups were compared using the SURVEYREG statement for continuous variables. Rao-Scott chi-square test was performed to examine differences in proportions between the groups using the SURVEYFREQ statement for categorical variables. Logistic regression was conducted using the SURVEYLOGISTIC statement to determine the associations between study variables and outcomes of interest. To balance the baseline characteristics between the groups (prior bariatric surgery or not), patients in the two groups were propensity score-matched (PSM). Propensity scores assigned to each patient were derived from the multivariable logistic regression model constructed to determine the likelihood of receiving bariatric surgery after controlling variables with a *p*-value < 0.01 in the unmatched population of the two groups. A 1:4 fixed ratio nearest-neighbor matching was performed. Further, univariate and multivariate logistic regression models were used to calculate the odds ratios (ORs) and 95% confidence intervals (CIs) in estimating the associations between study variables and patient outcomes. Variables that reached statistical significance in univariate analysis were included in multivariate models after adjusting for possible confounding variables. A two-sided *P*-value of < 0.05 was regarded as statistical significance.

## Results

### Study Sample

**Fig. 1:**
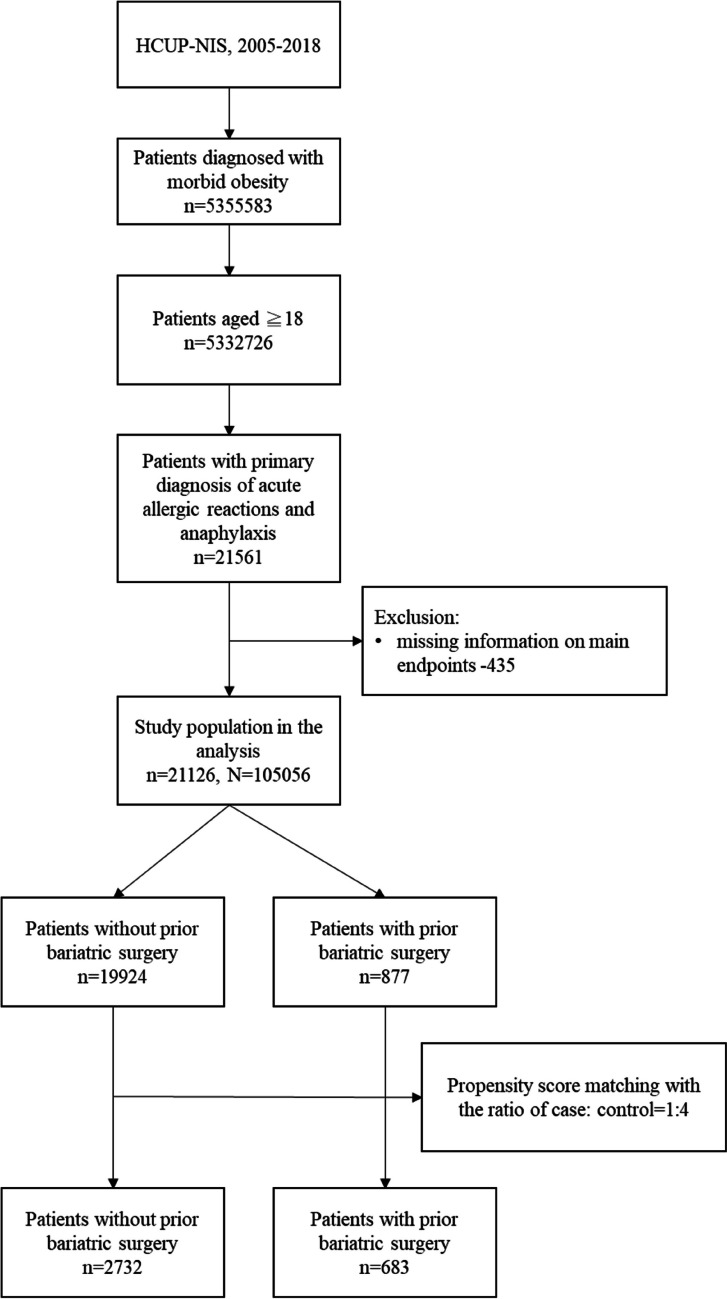
Flowchart of study sample selection

The study sample selection process is depicted in Fig. 1. A total of 21,561 hospitalized patients aged ≧18 diagnosed with morbid obesity and admitted to hospitals for acute allergic reactions and anaphylaxis were identified in the HCUP-NIS database of the US from 2005 to 2018. After excluding patients with missing information on LOS, discharge destination, in-hospital mortality, or hospital costs, 21,126 patients (representing a 105,056 US population) were finally included. Among these, 683 patients had undergone bariatric surgery, and 2,732 patients were not retained for subsequent analyses after 1:4 ratio PSM. Proportions of each primary diagnosis of acute allergic reaction and anaphylaxis are listed in Supplementary Table 2.

### Characteristics of Patients Hospitalized for Acute Allergic Reaction and Anaphylaxis

Supplementary Table 3 summarizes the characteristics of patients hospitalized for acute allergic reaction and anaphylaxis before PSM. The mean age of the Patients’ mean age was 54.7 ± 0.1 years, and females accounted for 67.5%. Significant differences were found between patients with or without prior bariatric surgery in age, sex, race, income, primary payer, some comorbidities (all *p* < 0.01), and hospital location. Patients with prior bariatric surgery were younger, with more females, and had fewer comorbidities than those without bariatric surgery. After PSM, the different initial distributions in the characteristics of patients and hospital between the two groups were balanced, as shown in Table 1.

**Table 1:** Characteristics of patients hospitalized for acute allergic reaction and anaphylaxis, after matching

	Overall (*N* = 16989) (*n* = 3415)	No prior bariatric surgery (*n* = 2732)	With prior bariatric surgery (*n* = 683)	*p*-value
Age	50.7 ± 0.2	51.0 ± 0.2	49.6 ± 0.5	**0.017**
18–29	160 (4.7)	119 (4.4)	41 (6.1)	0.105
30–39	584 (17.1)	458 (16.8)	126 (18.3)	
40–49	835 (24.5)	682 (25.0)	153 (22.5)	
50–59	893 (26.1)	704 (22.0)	189 (27.8)	
60–59	737 (21.6)	599 (22.0)	138 (20.1)	
70 +	206 (6.0)	170 (6.2)	36 (5.2)	
Sex				
Male	638 (18.6)	509 (18.6)	129 (18.8)	0.869
Female	2777 (81.4)	2223 (81.4)	554 (81.2)	
Race				
White	2511 (73.6)	2021 (74.1)	490 (72.0)	0.645
Black	591 (17.3)	463 (16.9)	128 (18.6)	
Hispanic	217 (6.3)	170 (6.2)	47 (6.7)	
Others	96 (2.8)	78 (2.8)	18 (2.6)	
Household income				
Q1	931 (27.2)	740 (27.0)	191 (28.2)	0.903
Q2	926 (27.2)	742 (27.3)	184 (26.9)	
Q3	913 (26.7)	735 (26.9)	178 (25.9)	
Q4	645 (18.9)	515 (18.8)	130 (19.0)	
Primary payer				
Medicare/Medicaid	1486 (43.4)	1200 (43.9)	286 (41.7)	0.129
Private including HMO	1735 (50.9)	1387 (50.8)	348 (51.1)	
Self-pay/no charge/others	194 (5.7)	145 (5.3)	49 (7.2)	
Admission type				
Elective	1211 (35.5)	964 (35.4)	247 (36.2)	0.708
Emergent	2204 (64.5)	1768 (64.6)	436 (63.8)	
Comorbidities				
CAD	308 (9.0)	248 (9.1)	60 (8.7)	0.756
Diabetes	1283 (37.5)	1032 (37.7)	251 (36.7)	0.590
Hypertension	1975 (57.9)	1583 (58.0)	392 (57.3)	0.745
Cerebrovascular disease	83 (2.4)	65 (2.4)	18 (2.6)	0.696
Chronic respiratory disease	1123 (32.9)	889 (32.5)	234 (34.2)	0.374
Rheumatic disease	106 (3.1)	88 (3.2)	18 (2.6)	0.381
Renal insufficiency	257 (7.5)	203 (7.4)	54 (7.9)	0.647
Hospital bedsize				
Large	1818 (57.9)	1458 (53.3)	360 (52.7)	0.965
Medium	1221 (35.7)	795 (29.2)	200 (29.4)	
Small	218 (6.4)	479 (17.5)	123 (17.8)	
Location/teaching status				
Urban teaching	1976 (57.9)	1591 (58.3)	385 (56.2)	0.578
Urban nonteaching	1221 (35.7)	968 (35.4)	253 (37.0)	
Rural	218 (6.4)	173 (68.3)	45 (6.8)	
Hospital region				
Northeast	562 (16.6)	432 (16.0)	130 (19.0)	0.345
Midwest	859 (25.2)	689 (25.2)	170 (25.1)	
South	1432 (41.9)	116 (42.3)	276 (40.3)	
West	562 (16.3)	455 (16.5)	107 (15.6)	

Continuous data are presented as mean ± SE; categorical data are presented as unweighted count (weighted %)

*CAD* coronary artery disease, *HMO* Health Maintenance Organization

### Outcomes of Patients Hospitalized for Acute Allergic Reaction and Anaphylaxis

Frequencies of outcomes among patients hospitalized for acute allergic reaction and anaphylaxis are listed in Table 2 and Supplemental Table 4. After matching, patients with prior bariatric surgery had a significantly lower proportion of any life-threatening morbidity (37.2% vs 47.4%), respiratory distress and failure (11.2% vs 17.0%), pneumonia and severe infection (7.4% vs 10.2%), sepsis and septic shock (15.2% vs 20.9%), intubation and mechanical ventilation (11.2% vs 14.6%), prolonged LOS (10.3% vs 20.6%) and unfavorable discharge (6.9% vs 12.5%) than those without prior bariatric surgery, respectively.

**Table 2:** Outcomes of patients hospitalized for acute allergic reaction and anaphylaxis, after matching

	After matching (*n* = 3415)		
	No prior bariatric surgery (*n* = 2732)	With prior bariatric surgery (*n* = 683)	*p*-value
Any life-threatening morbidity	1296 (47.4)	254 (37.2)	** < 0.001**
Cardiac arrest and dysrhythmia	281 (10.3)	54 (7.9)	0.056
AMI and CVA	91 (3.3)	19 (2.8)	0.399
VTE	122 (4.5)	22 (3.5)	0.190
Respiratory distress and failure	464 (17.0)	76 (11.2)	** < 0.001**
Pneumonia and severe infection	279 (10.2)	51 (7.4)	**0.019**
Sepsis and septic shock	571 (20.9)	104 (15.2)	** < 0.001**
Intubation and mechanical ventilation	400 (14.6)	77 (11.2)	**0.017**
Prolonged LOS	557 (20.6)	71 (10.3)	** < 0.001**
Unfavorable discharge	338 (12.5)	47 (6.9)	** < 0.001**
Mortality	29 (1.1)	3 (0.4)	0.129
Hospital cost	57,950 ± 1652.0	51,343 ± 2839.9	0.055

Continuous data are presented as mean ± SE; categorical data are presented as unweighted count (weighted %)

*AMI* acute myocardial infarction, *CVA* cerebrovascular accident, *VTE* venous thromboembolism, *LOS* length of stay

### Prior Bariatric Surgery and Outcomes in Patients Hospitalized for Acute Allergic Reaction and Anaphylaxis

A multivariable regression model was constructed to determine the associations between inpatient outcomes and prior bariatric surgery. The results are shown in Table 3. After adjusting for relevant confounders, patients who had prior bariatric surgery were significantly less likely to have life-threatening morbidity during hospitalization than those who had not (aOR:0.64, 95% CI:0.54–0.76). Also, patients with prior bariatric surgery had a significantly lower risk for prolonged LOS (aOR: 0.43, 95% CI:0.33–0.55). However, prior bariatric surgery was not associated with reduced odds of significant mortality or unfavorable discharge. Regarding individual morbidities, significantly lower odds were found in respiratory distress and failure (aOR: 0.61, 95% CI:0.47–0.78), pneumonia and severe infection (aOR: 0.71, 95% CI:0.53–0.96), sepsis and septic shock (aOR: 0.69, 95% CI:0.55–0.85), and intubation and mechanical ventilation (aOR: 0.72, 95% CI:0.56–0.93) in patients with prior bariatric surgery compared to those without prior bariatric surgery.

**Table 3:** Multivariate analysis of associations between prior bariatric surgery and inpatient outcomes

	With prior bariatric surgery (vs No prior bariatric surgery)
	aOR (95% CI)
Any life-threatening morbidity	**0.64 (0.54–0.76)**
Cardiac arrest and dysrhythmia	0.75 (0.56–1.02)
AMI and CVA	0.57 (0.30–1.08)
VTE	0.76 (0.51–1.14)
Respiratory distress and failure	**0.61 (0.47–0.78)**
Pneumonia and severe infection	**0.71 (0.53–0.96)**
Sepsis and septic shock	**0.69 (0.55–0.85)**
Intubation and mechanical ventilation	**0.72 (0.56–0.93)**
Prolonged LOS	**0.43 (0.33–0.55)**
Unfavorable discharge	0.51 (0.38–3.55)
Mortality	0.41 (0.12–1.39)

AMI, acute myocardial infarction; CAD, coronary artery disease; CVA, cerebrovascular accident; VTE, venous thromboembolism; LOS, length of stay

Multivariate models adjusted for:

Any morbidity: age, sex, race, primary payer, admission type, CAD, diabetes, cerebrovascular disease, chronic respiratory disease, renal insufficiency, location/teaching status

Cardiac arrest or dysrhythmia: age, sex, race, CAD, diabetes, hypertension, location/teaching status

AMI and CVA: age, sex, admission type, CAD, cerebrovascular disease, hospital bed size, location/teaching status

VTE: admission type, diabetes, hypertension

Respiratory distress and failure: age, sex, household income, primary payer, admission type, CAD, diabetes, cerebrovascular disease, chronic respiratory disease, renal insufficiency

Pneumonia and severe infection: age, sex, race, primary payer, admission type, hypertension, renal insufficiency

Sepsis and septic shock: age, sex, race, household income, admission type, CAD, diabetes, renal insufficiency, hospital bed size, location/teaching status, hospital region

Intubation and mechanical ventilation: age, gender, race, admission type, CAD, diabetes, cerebrovascular disease, chronic respiratory disease, renal insufficiency, hospital region

Prolonged LOS: age, primary payer, admission type, CAD, diabetes, cerebrovascular disease, renal insufficiency, hospital bed size

Unfavorable discharge: age, primary payer, diabetes, cerebrovascular disease, rheumatic disease, renal insufficiency, hospital bed size

Mortality: age, race, primary payer, CAD, cerebrovascular disease

## Discussion

The present study demonstrated that prior bariatric surgery is independently and significantly associated with a 36% lowered risk for any life-threatening morbidity and a 57% reduced risk for prolonged LOS in patients hospitalized for acute allergic reaction and anaphylaxis compared to those who did not undergo prior bariatric surgery. Lower risk was also observed for respiratory distress and failure, pneumonia and severe infection, sepsis and septic shock, and intubation and mechanical ventilation among patients who received prior bariatric surgery versus those who did not. To the best of our knowledge, the present study is the first to report the impact of bariatric surgery on outcomes after hospitalization for acute allergic reaction.

Anaphylaxis is classically defined as an allergen-driven process that induces specific IgE and activation of mast cells and basophils through cross-linking IgE receptors. However, it is also known that non-IgE-mediated pathways cause symptoms indistinguishable from those of classic anaphylaxis, and their activation may explain the severity of IgE-mediated anaphylaxis [15, 16]. A previous study demonstrated that obesity is a risk factor for neuromuscular blocking agent anaphylaxis, with risk increasing as body mass index (BMI) increases [17]. That result supports findings in the present study that weight loss from prior bariatric surgery in patients hospitalized for acute allergic reaction and anaphylaxis is associated with a lower risk for any life-threatening morbidity and prolonged LOS. Together, these results suggest that patients with a history of acute allergic reactions and anaphylaxis need strict control of weight, whether or not they have undergone bariatric surgery.

Although results of the present study indicate that patients with prior bariatric surgery are less likely to have life-threatening morbidity and prolonged LOS, the mechanism underlying the protective effect of bariatric surgery on outcomes of hospitalization for acute allergic reaction is unclear. A previous review article suggested that adipocytes, which store excess fuel in lipid vacuoles, may hypertrophy and eventually rupture, releasing internal contents that induce inflammatory processes [18]. Fat infiltrates adipose tissue and finds its way into the liver, skeletal muscle, pancreas, and other metabolically relevant organs; therefore, infiltrating adipose tissue into these organs results in local production of adipokines and pro-inflammatory cytokines. As adipose tissue organs expand with the infiltration of fat, the specific adipokines are produced and released into circulation at a higher rate, causing inflammation in other organ systems. Another study explained the link between obesity and food allergies based on the pro-inflammatory immunological effects of adipose tissue in obesity [19]. Consistent with the present research, a previous paper demonstrated that obese individuals had elevated total serum eosinophils and high IgE levels [20]. At the same time, in most people, anaphylaxis is caused by the presence of IgE to a specific allergen. Therefore, weight loss through bariatric surgery may reduce the severity of anaphylaxis through lowered circulating IgE levels.

## Strengths and Limitations

The present study is strengthened by the use of a large sample representing a nationwide population. The extended study period across 13 years also adds credence to the findings. PSM was also applied to minimize confounding of measured variables. However, this study is inherently limited by its retrospective and observational nature, which may limit the measurement of certain variables and generalization of findings to other populations. Selection bias also cannot be ruled out and the defined time periods in the database do not include follow-up data after discharge. Possible coding errors may have occurred as in other studies that used ICD code systems. Furthermore, we were unable to obtain data on patients’ responses to bariatric surgery, such as exact BMI changes and weight loss proportions, potentially limits further inference drawn from the analytical results. The precise severity of acute allergic reactions and comorbidities could not be distinguished because of insufficient data in the NIS. Medications prescribed were also not recorded in the database so these data could not be analyzed.

## Conclusions

Prior bariatric surgery predicts a lower risk of any life-threatening morbidity and prolonged LOS among adults hospitalized for acute allergic reaction and anaphylaxis. Further prospective studies incorporating measurements of exact weight loss following bariatric surgery remain necessary to substantiate the findings, and delve into the underlying mechanisms that elucidate the beneficial effects bariatric surgery within this patient population.

### Supplementary Information

Below is the link to the electronic supplementary material.

Supplementary file1 (DOCX 37.4 KB)

## Data Availability

All of the data supporting underlying findings are included in the manuscript and its supplemental files.
