# Supplementary material for: Prior Bariatric Surgery Predicts Lower Life-Threatening Morbidity in Patients Admitted for Acute Allergic Reaction and Anaphylaxis: a Propensity Score-Matched Analysis of the U.S. Nationwide Inpatient Sample, 2005–2018
Source: Obes Surg. 2024 Jul 24;34(9):3258–65. doi: 10.1007/s11695-024-07115-6 (PMC11349794; doi:10.1007/s11695-024-07115-6)
Supplement: Supplementary file 1 — Supplementary file1 (DOCX 37.4 KB) [file 11695_2024_7115_MOESM1_ESM.docx]

**Supplementary Table S1**. ICD codes for bariatric surgery, morbidity and allergic reactions/anaphylaxis

| Variables | ICD-9 | ICD-10 |
| --- | --- | --- |
| **Bariatric surgery** | Diagnosis: V45.86  Procedure: 44.31, 44.38, 43.39, 44.95, 43.82 | Diagnosis: Z98.84  Procedure: 0D1607A, 0D160JA, 0D160KA, 0D160ZA, 0D1687A, 0D168JA, 0D168KA, 0D168ZA, 0D16479, 0D1647A, 0D164J9, 0D164JA, 0D164K9, 0D164KA, 0D164Z9, 0D164ZA, 0DV64CZ, 0DB64Z3 |
| **Life-threatening morbidity** |  |  |
| Cardiac arrest and dysrhythmia | Diagnosis: 427 | Diagnosis: I46 |
| AMI | Diagnosis: 410  DXCCSn=100 | Diagnosis: I21, I22  DXCCSR_CIR009>0 |
| CVA | Diagnosis: 433.01, 433.10, 433.11, 433.21, 433.31, 433.81, 433.91, 434.00, 434.01, 434.11, 434.91, 436, 430, 431  DXCCSn=109 | Diagnosis: I60, I61, I62, I63, I64, I69, G45, G46  DXCCSR_CIR020>0 |
| VTE | Diagnosis: 415, 451-453, 671, 673, 997.2 | Diagnosis: I260, I269, I801-803, I808, I809, I820-I823, I828, I829, O082, O223, O871, O882, I81, I82 |
| Respiratory distress and failure | Diagnosis: 799.1, 518.8 | Diagnosis: J96, J95.2-J95.8, J80, J81.0 |
| Pneumonia | Diagnosis: 486, 481, 482.8, 482.3 | Diagnosis: A48.1, J12 - J18 |
| Infection | Diagnosis: 518.6, 686, 136 | Diagnosis: L00-L08, B99, B44.81 |
| Sepsis and septic shock | Diagnosis: 995.9, 996.64, 038, 999.3, 790.7, 041, 785.52, 995.0 | Diagnosis: R78.81, A41, R65.2, T81.4, T80.2, A42.7, A22.7, B37.7, A26.7, A28.2, A54.86, B00.7, A32.7, A24.1, A39.2, A20.7, A21.7, A48.3, A40, R65.1, T78.2 |
| Intubation and mechanical ventilation | Diagnosis: V46.1, 495.7,  Procedure: 93.9, 96.7, 96.01-96.05 | Diagnosis: Z99.1, J95.85  Procedure: 5A190, 5A193, 5A194, 5A195, 5A093, 5A094, 5A095, 0BH1 |
| **Allergic reactions and anaphylaxis** |  |  |
| Anaphylactic reaction due to food | Diagnosis: 995.6 | Diagnosis: T78.0 |
| Other adverse food reactions | Diagnosis: 995.7 | Diagnosis: T78.1 |
| Anaphylactic shock | Diagnosis: 995.0 | Diagnosis: T78.2 |
| Angioneurotic edema | Diagnosis: 995.1 | Diagnosis: T78.3 |
| Allergy, unspecified | Diagnosis: 995.3 | Diagnosis: T78.4 |
| Anaphylactic reaction due to serum | Diagnosis: 999.4 or 999.4 | Diagnosis: T80.5 |
| Anaphylactic reaction due to adverse effect of correct drug or medicament properly administered | Diagnosis: 995.27 | Diagnosis: T88.6 |
| Unspecified adverse effect of drug or medicament | Diagnosis: 995.2 | Diagnosis: T88.7 |
| Urticaria | Diagnosis: 708 | Diagnosis: L50 |
| Eosinophilia | Diagnosis: 288.3 | Diagnosis: D72.1 |

AMI, acute myocardial infarction; CVA, cerebrovascular accident; VTE, venous thromboembolism.

**Supplementary Table S2.** Frequency of each primary diagnosis of hospitalization for acute allergic reactions and anaphylaxis

| **Primary diagnosis** | Numbers |
| --- | --- |
| Anaphylactic reaction due to food | 446 |
| Other adverse food reactions | 269 |
| Anaphylactic shock | 1724 |
| Angioneurotic edema | 6776 |
| Allergy, unspecified | 2109 |
| Anaphylactic reaction due to serum | 74 |
| Anaphylactic reaction due to adverse effect of correct drug or medicament properly administered | 1017 |
| Unspecified adverse effect of drug or medicament | 1962 |
| Urticaria | 5067 |
| Eosinophilia | 3424 |

**Supplementary Table S3**. Characteristics of patients hospitalized for acute allergic reaction and anaphylaxis, before matching

|  | Overall  (n=21126)  (N=105056) | No prior bariatric surgery  (n=19924) | With prior bariatric surgery  (n=877) | p-value |
| --- | --- | --- | --- | --- |
| **Age** | 54.7 ± 0.1 | 55.0 ± 0.1 | 48.5 ± 0.4 | **<0.001** |
| 18-29 | 1174 (5.6) | 1124 (5.5) | 50 (5.7) | **<0.001** |
| 30-39 | 2300 (10.9) | 2123 (10.5) | 177 (20.1) |  |
| 40-49 | 3938 (18.6) | 3704 (18.3) | 234 (26.6) |  |
| 50-59 | 5336 (25.3) | 5107 (25.2) | 229 (26.1) |  |
| 60-59 | 4969 (23.5) | 4816 (23.8) | 153 (17.4) |  |
| 70+ | 3409 (16.1) | 3372 (16.7) | 37 (4.2) |  |
| **Sex** |  |  |  |  |
| Male | 6871 (32.5) | 6723 (33.2) | 148 (16.8) | **<0.001** |
| Female | 14251 (67.5) | 13519 (66.8) | 732 (83.2) |  |
| Missing | 4 | 4 | 0 |  |
| **Race** |  |  |  |  |
| White | 11931 (61.0) | 11327 (60.4) | 604 (74.5) | **<0.001** |
| Black | 5173 (26.5) | 5039 (26.9) | 134 (16.4) |  |
| Hispanic | 1717 (8.8) | 1666 (8.9) | 51 (6.1) |  |
| Others | 743 (3.8) | 719 (3.8) | 24 (3.0) |  |
| Missing | 1562 | 1495 | 67 |  |
| **Household income** |  |  |  |  |
| Q1 | 7154 (34.6) | 6938 (35.0) | 216 (25.1) | **<0.001** |
| Q2 | 5571 (26.9) | 5326 (26.8) | 245 (28.3) |  |
| Q3 | 4805 (23.2) | 4569 (23.0) | 236 (27.3) |  |
| Q4 | 3193 (15.4) | 3026 (15.2) | 167 (19.3) |  |
| Missing | 403 | 387 | 16 |  |
| **Primary Payer** |  |  |  |  |
| Medicare/Medicaid | 12392 (58.8) | 12072 (59.8) | 320 (36.5) | **<0.001** |
| Private including HMO | 6810 (32.3) | 6315 (31.2) | 495 (56.9) |  |
| Self-pay/no charge/others | 1875 (8.9) | 1817 (9.0) | 58 (6.5) |  |
| Missing | 49 | 42 | 7 |  |
| **Admission type** |  |  |  |  |
| Elective | 2858 (13.6) | 2458 (87.8) | 400 (45.7) | **<0.001** |
| Emergent | 18208 (86.4) | 2458 (12.2) | 478 (54.3) |  |
| Missing | 60 | 58 | 2 |  |
| **Comorbidities** |  |  |  |  |
| CAD | 3765 (17.8) | 3700 (18.3) | 65 (7.3) | **<0.001** |
| Diabetes | 10002 (47.3) | 9708 (47.9) | 294 (33.3) | **<0.001** |
| Hypertension | 13307 (63.0) | 12832 (63.4) | 475 (53.9) | **<0.001** |
| Cerebrovascular disease | 894 (4.2) | 876 (4.3) | 18 (2.0) | **<0.001** |
| Chronic respiratory disease | 8403 (39.8) | 8108 (40.1) | 295 (33.5) | **<0.001** |
| Rheumatic disease | 608 (2.9) | 583 (2.9) | 25 (2.8) | 0.900 |
| Renal insufficiency | 3835 (18.2) | 3776 (18.7) | 59 (6.7) | **<0.001** |
| **Hospital bed size** |  |  |  |  |
| Large | 11877 (56.4) | 11421 (56.6) | 456 (52.0) | 0.066 |
| Medium | 5857 (27.9) | 5604 (27.8) | 253 (29.0) |  |
| Small | 3334 (15.7) | 3165 (15.6) | 169 (19.0) |  |
| Missing | 58 | 56 | 2 |  |
| **Location/teaching status** |  |  |  |  |
| Urban teaching | 11903 (56.5) | 11399 (56.5) | 504 (57.2) | **0.0049** |
| Urban nonteaching | 6971 (33.1) | 6653 (32.9) | 318 (36.3) |  |
| Rural | 2194 (10.4) | 2138 (10.6) | 56 (6.5) |  |
| Missing | 58 | 56 | 2 |  |
| **Hospital region** |  |  |  |  |
| Northeast | 3129 (14.9) | 2973 (14.8) | 156 (17.7) | 0.072 |
| Midwest | 5718 (27.1) | 5462 (27.0) | 256 (29.4) |  |
| South | 8822 (41.7) | 8481 (41.9) | 341 (38.5) |  |
| West | 3457 (16.3) | 3330 (16.3) | 127 (14.3) |  |

**Continuous data are presented as mean** ± SE; categorical data are presented as unweighted count (weighted %).

CAD, coronary artery disease; HMO, Health Maintenance Organization

**Supplementary Table S4.** Outcomes of patients hospitalized for acute allergic reaction and anaphylaxis, before matching.

|  | Before matching (n=21126) | | |
| --- | --- | --- | --- |
|  | No prior bariatric surgery  (n=19924) | With prior bariatric surgery  (n=877) | p-value |
| **Any life-threatening morbidity** | 11276 (55.7) | 297 (33.7) | **<0.001** |
| Cardiac arrest and dysrhythmia | 2752 (13.6) | 62 (7.0) | **<0.001** |
| AMI and CVA | 1011 (5.0) | 8 (0.9) | **<0.001** |
| VTE | 895 (4.4) | 30 (3.6) | 0.257 |
| Respiratory distress and failure | 4513 (22.3) | 86 (9.8) | **<0.001** |
| Pneumonia and severe infection | 2606 (12.9) | 37 (4.1) | **<0.001** |
| Sepsis and septic shock | 4526 (22.3) | 124 (14.0) | **<0.001** |
| Intubation and mechanical ventilation | 3789 (18.7) | 90 (10.2) | **<0.001** |
| **Prolonged LOS** | 4627 (23.2) | 92 (10.4) | **<0.001** |
| **Unfavorable discharge** | 3004 (15.1) | 55 (6.2) | **<0.001** |
| **Mortality** | 322 (1.6) | 3 (0.3) | **0.003** |
| **Hospital cost** | 57959 ± 758.3 | 50922 ± 2476.0 | **0.006** |

**Continuous data are presented as mean** ± SE; categorical data are presented as unweighted count (weighted %).

AMI, acute myocardial infarction; CVA, cerebrovascular accident; VTE, venous thromboembolism; LOS, length of stay.
